# What is new in vascular dementia?

**DOI:** 10.1186/s12916-016-0726-z

**Published:** 2016-11-03

**Authors:** Amos D. Korczyn

**Affiliations:** Department of Neurology, Tel Aviv University, Ramat Aviv, 69978 Israel

**Keywords:** Alzheimer’s, Cognition, Dementia, Vascular

## Abstract

Any damage to the brain may affect cognition. However, although the effects of vascular changes have been known for years, involvement of such changes is becoming increasingly better recognized. In particular the effects of comorbid vascular disease to primary neurodegenerative processes adds to the complexity of the issue. An attempt to clarify the problems needed specific attention to different points, which led to Consensus Reports on several of them.

## Editorial

The ever-increasing global healthcare burden of dementia [1] has changed considerably over the past decades. Although Alzheimer’s disease (AD) is still considered the most common disorder, it’s nosological entity is undergoing constant revision. This is owing to the recognition that the underlying brain pathology in most if not all elderly people is complex and includes widespread vascular changes, as well as deposition of TDP-43 and other abnormal constituents. Vascular dementia (VaD) was previously considered the second most common type of dementia, but has been superseded by two other disorders, frontotemporal dementia and Lewy body disease.

Of these four disorders, most attention and efforts have been devoted to AD, alas so far without impressive therapeutic results [2]. Therapies for the neurodegenerative diseases frontotemporal dementia and Lewy body disease have also not yet been determined, nor is a breakthrough evident. However, vascular damage to the brain has a clear pathogenesis and thus makes a much more likely therapeutic target.

Vascular cognitive impairment (VCI) is a blanket term that covers a wide spectrum of disorders. They range in intensity from mild cognitive impairment to full-blown dementia, and in presentation from acute cortical strokes to subcortical lesions, either lacunes or white matter disease (or both in combination). Their pathogenesis can range from vascular occlusions to hemorrhages, and their cause can be genetic (such as cerebral autosomal-dominant arteriopathy with subcortical infarcts and leukoencephalopathy, CADASIL), atherosclerotic, embolic, hypoperfusion, and others.

The International Congress of Vascular Dementia (ICVD) is a biannual convention that began in 1999 and was last held in Ljubljana, Slovenia, in October 2015. During this meeting, several teams of experts, coming from different backgrounds and geographical origins, were formed to tackle problems in the diagnosis and treatment of vascular dementia. Their aim was to produce consensus statements on some of the contentious topics within the field. Some of these teams have now been successful in formulating conclusions, which are now published in *BMC Medicine* [3–6].

Clinical diagnostic criteria for VCI are still not satisfactory. An attempt to clarify the confusion and delineate future solutions to existing needs was made by Perneczky et al. [3]. Modern neuroimaging techniques using magnetic resonance imaging (MRI) are very sensitive for showing white matter changes, lacunes, and microbleeds, and their contribution is discussed by Heiss et al. [4].

In many disorders, autopsy is the gold standard for diagnosis. However, in cognitive disorders there are several impediments to a dependence on pathology. One important problem is the heterogeneity of the brain lesions, mentioned above. Another is the wide spectrum of neuropsychological manifestations. But perhaps most problematic is the fact that pathologists can diagnose that the brain has been damaged by vascular lesions, but cannot determine the clinical correlate. Thus, people with the same extent of apparent brain damage may have different cognitive manifestations. Moreover, the additional damage imposed by other pathologies, such as the common co-occurrence of AD pathology [7], complicates the interpretation of the pathological findings. An attempt to deal with these issues and suggest solutions has been made by McAleese et al. [5].

White matter disease of the brain is commonly seen in elderly people and is commonly caused by small vessel disease. However, the clinical manifestations of these changes are again heterogeneous. In some cases, patients may exhibit VCI up to severe dementia, which may be termed Binswanger’s disease, others may have gait problems or vascular parkinsonism [8], while still others develop depression. The entity of vascular depression is the topic of a consensus statement by Aizenstein et al. [6].

These articles are an important contribution to the existing literature. Surely further additions will appear in the following months, and even more after the tenth ICVD congress, scheduled to take place in Gdansk, Poland, on 12–14 May 2017 (www.ICVD2017.pl).

## References

[1] Wimo A, Guerchet M, Ali GC, Wu YT, Prina AM, Winblad B, Jönsson L, Liu Z, Prince M (2016). The worldwide costs of dementia 2015 and comparisons with 2010. Alzheimers Dement.

[2] Korczyn AD (2012). Why have we failed to cure Alzheimer's disease?. J Alzheimers Dis.

[3] Perneczky R, Tene O, Attems J, Giannakopoulos P, Ikram MA, Federico A, Sarazin M, Middleton LT (2016). Is the time ripe for new diagnostic criteria of cognitive impairment due to cerebrovascular disease? Consensus report of the International Congress on Vascular Dementia working group. BMC Med.

[4] Heiss WD, Rosenberg GA, Thiel A, Berlot R, De Reuck J (2016). Neuroimaging in vascular cognitive impairment: a state-of-the-art review. BMC Med.

[5] McAleese KE, Alafuzoff I, Charidimou A, De Reuck J, Grinberg LT, Hainsworth AH, Hortobagyi T, Ince P, Jellinger K, Gao J, Kalaria RN, Kovacs GG, Kövari E, Love S, Popovic M, Skrobot O, Taipa R, Thal DR, Werring D, Wharton SB, Attems J (2016). Post-mortem assessment in vascular dementia: advances and aspirations. BMC Med.

[6] Aizenstein HJ, Baskys A, Boldrini M, Butters MA, Diniz BS, Jaiswal MK, Jellinger KA, Kruglov LS, Meshandin IA, Mijajlovic MD, Niklewski G, Pospos S, Raju K, Richter K, Steffens DC, Taylor WD, Tene O (2016). Vascular depression consensus report – a critical update. BMC Med.

[7] Korczyn AD (2002). Mixed dementia--the most common cause of dementia. Ann N Y Acad Sci.

[8] Korczyn AD (2015). Vascular parkinsonism--characteristics, pathogenesis and treatment. Nat Rev Neurol.

